# Boarding Injuries: The Long and the Short of It

**DOI:** 10.1155/2014/924381

**Published:** 2014-02-10

**Authors:** Leslie A. Fabian, Steven M. Thygerson, Ray M. Merrill

**Affiliations:** ^1^Utah Valley Regional Medical Center, Provo, UT 84604, USA; ^2^Department of Health Science, Brigham Young University, 229-C Richards Building, Provo, UT 84602, USA

## Abstract

As the popularity of longboarding increases, trauma centers are treating an increased number of high severity injuries. Current literature lacks descriptions of the types of injuries experienced by longboarders, a distinct subset of the skateboarding culture. A retrospective review of longboarding and skateboarding injury cases was conducted at a level II trauma center from January 1, 2006, through December 31, 2011. Specific injuries in addition to high injury severity factors (hospital and intensive care unit (ICU) length of stay (LOS), Injury Severity Score (ISS), patient treatment options, disposition, and outcome) were calculated to compare longboarder to skateboarder injuries. A total of 824 patients met the inclusion criteria. Skull fractures, traumatic brain injuries (TBI), and intracranial hemorrhage (ICH) were significantly more common among longboard patients than skateboarders (*P* < 0.0001). All patients with an ISS above 15 were longboarders. Hospital and ICU LOS in days was also significantly greater for longboarders compared with skateboarders (*P* < 0.0001). Of the three patients that died, each was a longboarder and each experienced a head injury. Longboard injuries account for a higher incidence rate of severe head injuries compared to skateboard injuries. Our data show that further, prospective investigation into the longboarding population demographics and injury patterns is necessary to contribute to effective injury prevention in this population.

## 1. Introduction

Since the advent of the skateboard in the late 1950's, skateboarding has moved from a subculture of surfing to a mainstream recreational activity. There are no definitive numbers that quantify longboarding's growth, but manufacturers' steady increase in sales indicates the growing popularity in longboarding. Shortly following in the 1960's, longboarding gained increasing popularity throughout both the United States and the world. This popularity of longboards and their associated injuries has created a need to better define and understand the sport. While longboarding is commonly classified with skateboarding, it is a distinct variation of the latter, unique not only in the board dimensions and engineering but also in function. Longboards are generally 42 to even 80 inches in length, compared with regular skateboards that are 30 to 38 inches long. The longer length and increased width of the longboard allow for greater travel, higher speeds, downhill cruising, and carving. The current world record for the fastest speed is held at 80.74 mph, set on June 18, 2012 [1]. While these professional speeds are not maintained by lay longboarders, they may reach speeds up to 30+ mph depending on the terrain.

Due to these characteristics, longboarding serves as a cross-training outlet among surfers and snowboarders alike during off-seasons because of the similar ride and movement of the vehicles. As the popularity of longboards increases, undoubtedly the injuries that accompany their use will also increase.

Studies on skateboarding injuries have been well documented [2–5]. However, there is a paucity of corresponding data with regards to longboarding injuries. In light of the defined difference in use between longboards and skateboards, we hypothesize that longboarding lends to more severe injuries than regular skateboarding injuries. The purpose of this study is to derive baseline data regarding longboarding injuries and compare said data to that relating to skateboard-specific injuries.

## 2. Methods

A retrospective review of longboarding and skateboarding injury cases was conducted at a level II trauma center from January 1, 2006 through December 31, 2011. Patients were identified from emergency department (ED) records and hospital trauma registry using the external cause of injury code (E-code) of the International Classification of Diseases—9th Revision specific for “fall from skateboard” (885.2). Records were then reviewed individually by a single abstractor to distinguish the vehicle of interest of each case. Inclusion criteria consisted of all records in which acute injuries were sustained while longboarding or skateboarding. Misclassified cases in which some other vehicle was implicated (bicycle, rollerblades, etc.) were excluded. It was noted in the abstraction process that in 49 cases different practitioners documented on the same record contradicted each other in their use of “skateboard” and “longboard.” In such cases, the more specific classification of longboard was used. The age and sex distribution was not significantly different between the longboarding group (without these 49 patients) compared with these 49 patients (data not shown).

Demographic information extracted from the records included age, sex, the etiology of the injury (e.g., hit a car, collided with another skater, or isolated fall), helmet use, and patient outcome. Diagnosis was obtained from documented clinical impressions and supplemented by independent review of the history, physical examination, and diagnostic studies performed. Mild traumatic brain injury (TBI) was defined as Glasgow Coma Score (GCS) 13–15 without focal neurological findings and no findings on neuroimaging. Moderate TBI was defined by the presence of one of the following: GCS 9–12, positive findings on neuroimaging, and/or focal neurologic finding. Severe TBI was differentiated from moderate TBI by a GCS of 3–8. Other information obtained from the records included whether head impact occurred, the intervention taken, and the disposition of the patient. Head fractures, TBI, intracranial hemorrhage (ICH), hospital and ICU length of stay (LOS), ISS, patient disposition, and outcome were selected as severity indicators. Data were deidentified to maintain the patient confidentiality. This study was approved by the institutional review boards of Intermountain Healthcare and Brigham Young University.

Basic summary statistics were used to describe the data, including means, standard deviations, and percentages. Bivariate analyses involving longboard/skateboard status and selected nominal or grouped variables were assessed using the chi-square test. Fisher's exact test was also used when small numbers were involved. Differences in means were evaluated using linear regression analysis and the *F* test. Rate ratios were calculated using Poisson regression. Both regression models included age and sex in order to adjust for these variables. Statistical significance of these rate ratios was evaluated using 95% confidence intervals; that is, significance was present if the confidence interval did not overlap 1. Two-sided tests of hypotheses were evaluated using the 0.05 level of significance. Computations were performed using the Statistical Analysis System (SAS) software, version 9.3 (SAS Institute Inc., Cary, NC, USA, 2010).

## 3. Results

A total of 824 patients (146 in 2006, 143 in 2007, 149 in 2008, 112 in 2009, 146 in 2010, and 128 in 2011) met the inclusion criteria. Patients ranged in age from 2 to 48 (*M* = 19.2, SD = 6.1), and 75.2% were male. Most accidents occurred in Utah County (91.0%, 2.4% in surrounding counties, and 6.6% unknown). Only 2.0% of injuries involved a vehicle and 1.9% involved collision with another boarder. The majority of reported accidents involved longboards (57.5%) compared with skateboards (42.5%).

The association between longboard versus skateboard status and selected variables is presented in Table 1. Compared with skateboard patients, longboard patients were significantly older and female. The large percentage of unknown information about helmet use limits any conclusion about this variable.

Injuries were classified into several categories (Table 2). Dermal injuries and fractures were the most common types of injury, followed by traumatic brain injuries. Extremity fractures (particularly involving the clavicle) and dermal injuries were significantly more common among longboard patients. On the other hand, soft tissue injuries involving the lower extremity were significantly less common among longboard patients.

Head fractures, spine and traumatic brain injury, and intracranial hemorrhage are further presented according to longboard/skateboard status (Table 3). The table also shows ICU and hospital length of stay, injury severity scores, and disposition of patients and whether they survived the accident. Head fractures, traumatic brain injuries, and intracranial hemorrhage were significantly more common among longboard patients. All patients with an injury severity score (ISS) above 15 were longboarders (6, 1.2% with a score of 16–24, and 7, 1.4% with a score of 25+). The ISS was not significantly associated with either age (*F* = 0.05, *P* = 0.4722) or sex (*F* = 0.28, *P* = 0.7534). Length of stay (LOS) in days was also significantly greater for longboarders compared with skateboarders (*M* = 0.52, SD = 1.76 versus *M* = 0.11, SD = 0.57; *F* = 17.70, *P* < 0.0001). Length of stay was not significantly associated with age (*F* = 2.10, *P* = 0.1478) or sex (*F* = 1.64, *P* = 0.2009). Head impact was documented in 309 (37.9%) of all patients. Longboarders were significantly more likely to have a head impact than skateboarders (49.0% versus 21.7%, Chi-square = 92.9, *P* < 0.0001). Of the three patients that died, each one was a longboarder and experienced a head injury. Two of the three deaths were isolated (non-motor-vehicle collision injuries) and the third was unknown etiology.

The frequency of patient treatments according to longboard/skateboard status is presented in Table 4. Approximately 8.1% underwent an operation, primarily orthopedic surgery. Longboarders were significantly more likely to undergo surgery overall and neurosurgery in particular. Age and sex were not significantly associated with conservative treatment or neurosurgical intervention after adjusting for longboard/skateboard status. Sex was not significantly associated with orthopedic surgical intervention, but the frequency of this treatment increased with age (3.5% for ages < 10, 5.7% for ages 10–24, and 13.6% for ages 25+).

The effect of age on fractures and traumatic brain injury according to longboard/skateboard status is presented in Table 5. Among longboard patients, the incidence of radius/ulna/radial head was significantly more common in patients less than 15 years and the incidence of mild traumatic brain injury was significantly greater for ages 15–24 year. Among skateboarding patients, the incidence of upper extremity fractures and fractures involving the radius/ulna/radial head was significantly greater among ages less than 15, whereas lower extremity fractures and fractures involving the tibia/fibula/ankle were significantly lower among ages less than 15. The incidence of lower extremity fractures increased with age.

Being female was associated with increased risk of lower extremity fractures (12.4% versus 5.3%, *P* = 0.0062) among longboard patients and tibia/fibula/ankle fractures (11.8% versus 3.3%, *P* = 0.0078) among skateboard patients. Sex was not significantly associated with the other types of injuries.

## 4. Discussion

Current literature reveals a paucity of data with regard to longboarding and its accompanying injuries. While the two most frequent injuries among longboarders, namely, dermal injuries and extremity fractures, are clinically underwhelming, the frequency of TBI and skull fractures among longboarders versus skateboarders is the most notable among clinical outcomes. Injuries of such high severity begin to distinguish longboarder injuries. Though skateboards, like longboards, can be used on the street as a form of transportation, they are associated most commonly with skate parks and other venues allowing for acrobatic tricks and less for attainment of significant speeds. However, the usage of longboards for transportation, higher speeds, and downhill travel, place riders at an increased risk of TBI.

Lustenberger et al. conducted a study to evaluate skateboard-related epidemiology using five years of skateboard-injury data from the National Trauma Databank [6]. Despite differences in definition of TBI (skull fracture was included in the “Overall TBI” definition in the Lustenberger study), our longboard-specific TBI incidence of 30.6% (38.6% with skull fractures factored in) is comparable to their numbers of 36.3%. Contrastingly, our skateboard-specific numbers varied significantly at 12.7% (12.9% with skull fractures factored in). The Lustenberger study also found a positive association between age and TBI [6]. Similarly, we found that longboard patients had an older age distrubtion. Lustenberger et al. did not distinguish between longboards and skateboards; however, the current study indicates that longboard status should be a variable of skateboard-associated TBI.

Tominaga et al. found a positive association between age and severity of injury among skateboarders. Patients in their study were older (70% at least 18 years), and injuries tended to involve the head, with neurosurgical intervention frequently required [7]. While their study did not differentiate between skateboarders and longboarders, it raises the question as to whether the apparent increased frequency of head injury relates directly to patient age or if older patients are more likely to be longboarders. As found in the current study, longboarders were more likely to experience head injuries and were of older age.

In a study investigating the characteristic features of snowboard head injuries, Nakaguchi et al. found that, of 559 snowboard-injury related patients during the study period, 26% sustained a head injury characterized by symptoms such as transient amnesia, headache, concussion, nausea, and/or open wound [8]. While this definition prevents a fair comparison to the data in our study, they found that 6.3% of snowboard head injuries could be classified as “major head injury” or head injury with positive CT scan findings such as intracranial bleeding or brain edema [9]. These numbers correspond with our findings of (similarly defined) moderate to severe traumatic brain injuries (6.2% and 1.7%, resp.) in our longboard-associated injury patients. This may relate to similarities in the two sports including stance, downhill speeds, and even terrain as the Nakaguchi study emphasized that the slopes of Japan often consist of icy, hard-packed snow, which may be similar to firm ground encountered in longboarding [8].

Extensive investigation into helmet use and prevention of TBI among skiers/snowboarders has been conducted over the years. This has recently prompted a practice management guideline established by the Eastern Association for the Surgery of Trauma which recommends the use of safety helmets to reduce the severity and incidence of head injuries during these activities [9]. In light of the similarities between snowboarding and longboarding and their resulting head injuries, this same recommendation may correspond to longboarding, though further research is indicated. Unfortunately, according to our data, almost half of our longboarding population (44.6%) was documented as *not* wearing a helmet while only 4.1% took this precaution. The remaining 51.2% had no documentation leaving the actual frequency of helmet use in question.

In addition to the retrospective nature of this study, it is limited by its confinement to a single trauma center. Because the trauma center of interest serves such a large geographic area, a selection bias towards more severely injured patients may result as such would be transferred to the center for definitive care. Patients with less severe injuries treated and discharged from surrounding hospitals are not accounted for nor are those longboarders/skateboarders who did not seek treatment for their injuries. Additionally, data with regards to chemical impairment, boarding conditions (lighting, weather, etc.), and boarder experience were markedly limited preventing us from factoring these variables into the data.

The actual number of longboarding incidents remains difficult to ascertain. While the number in this study may arguably have been overestimated by our inclusion of dual-labeled accidents with the longboarding population since it was the more specific definition, this accounted for only 6% of the included population. This trend of contradictory labels may be the result of unfamiliarity with the sport and potentially lead to an underestimation of the longboard population due to inability to distinguish the two sports from one another.

While the results of this study should not be misconstrued to imply that skateboarders do not need to wear helmets, it does demonstrate the existence of an at-risk population not previously identified in the literature. These results may help better focus on injury prevention resources such as education and public health interventions. Campaigns promoting helmet use, while pertinent to both skateboarders and longboarders, may significantly reduce TBI and head fractures when customized to the longboarding population. Continued helmet use by longboarding professionals, promotion of helmet use by local longboarding shops, local helmet use promotion events, and discounted or free distribution of helmets to longboarders are methods that may increase the use of helmets while longboarding. Health care professionals should make it a priority to inform the skateboarding and longboarding population about the importance of helmet use.

## 5. Conclusion

Like skateboarding, longboarding, a unique variant of the former, has been climbing in popularity in recent years. While there is a fair amount of research regarding skateboarding and its accompanying injury patterns and demographics, there is a noted absence of data regarding longboarding. In this study, we have found distinct variations both in population demographics and injury patterns between the two groups with longboarding demonstrating an increased tendency toward life altering or ending injuries. It is widely accepted that many of these higher-velocity injuries, head injuries in particular, can be easily prevented through proper helmet use and education.

For emergency medicine practitioners, distinguishing between longboard and skateboard on history may help direct the patient work-up. While the signs and symptoms of TBI may be difficult to clinically overlook, our data show that a higher-than-normal index of suspicion should be maintained when caring for a longboard-injury patient, leading to a lower threshold for neuroimaging studies. Future development of a longboard specific E-code may promote a distinction between the two sports. While the results of this study should not be misconstrued to imply that skateboarders do not need to wear helmets they may help better focus on education and public health interventions. Campaigns promoting helmet use, while pertinent to both skateboarders and longboarders, may significantly reduce TBI and head fractures when customized to the longboarding population.

Further research is required to better understand this population and injury patterns so that appropriate preventative measures may be publicized.

## Figures and Tables

**Table 1:** Longboard/skateboard status according to selected variables.

	Longboard		Skateboard		Chi-square
	(*n* = 474)		(*n* = 350)		*P* value
	Number	%	Number	%	
Age					
2–9	3	0.4	27	7.7	<0.0001
10–14	19	4.0	115	32.9	
15–19	190	40.1	116	33.1	
20–24	194	40.9	51	14.6	
25+	69	14.6	41	11.7	
Sex					
Male	321	67.7	299	85.4	<0.0001
Female	153	32.3	51	14.6	
Etiology					
Isolated	447	94.3	338	96.6	0.2355
Vehicle	13	2.7	4	1.1	
Other	9	1.9	7	2.0	
Unknown	5	1.1	1	0.3	
Helmet use					
Yes	19	4.0	6	1.7	<0.0001
No	216	45.6	46	13.2	
Unknown	239	50.4	298	85.1	

Note: the percent column sum is 100 for each variable.

**Table 2:** Injury types according to longboard/skateboard status.

Specific injuries	Longboard		Skateboard		Chi-square	Rate ratio*	95% CI*
	(*n* = 474)		(*n* = 350)		*P* value		
	Number	%	Number	%			
Extremity fractures	191	40.3	125	35.7	0.1813	1.09	0.99–1.21
Upper extremity	112	23.6	100	28.6	0.1086	1.04	0.92–1.17
Radius/ulna/radial head	53	11.2	66	18.9	0.0019	0.90	0.75–1.07
Clavicle (collarbone)	39	8.2	8	2.3	0.0003	1.40	1.24–1.57
Wrist/hand	17	3.6	11	3.1	0.7283	1.13	0.88–1.45
Humerus	2	0.4	8	2.3	0.0153^†^	0.60	0.21–1.77
Lower	36	7.6	22	6.3	0.4677	0.90	0.74–1.10
Femur	5	1.0	3	0.9	0.7748	0.90	0.55–1.47
Tibia/fibula/ankle	21	4.4	16	4.6	0.9231	0.84	0.64–1.10
Soft tissue injury							
Upper extremity	75	15.8	59	16.9	0.6908	0.99	0.86–1.15
Lower extremity	53	11.2	61	17.4	0.0102	0.78	0.66–0.93
Head/face	22	4.6	7	2.0	0.0420	1.11	0.93–1.34
Neck/back	16	3.4	10	2.9	0.6739	1.03	0.77–1.37
Chest/abdomen	4	0.8	1	0.3	0.3078		
Dermal injury	221	46.6	71	20.3	<0.0001	1.42	1.29–1.56
Dislocation	19	4.0	12	3.4	0.6654	1.03	0.80–1.32
Pulmonary injury^‡^	3	0.6	0	0.0	0.1359		
Solid organ injury	4	0.8	0	0.0	0.0849		

*Risk of the injury for longboarder compared with skateboarder patients, adjusted for age and sex.

^†^Based on Fisher's Exact Test.

^‡^Pneumothorax, pulmonary contusion.

**Table 3:** Head fractures, spinal injuries, traumatic brain injury, and hemorrhages, along with selected outcomes according to longboard/skateboard status.

Specific injuries	Longboard		Skateboard		Chi-square	Rate ratio*	95% CI*
	(*n* = 474)		(*n* = 350)		*P* value		
	Number	%	Number	%			
Head fractures	41	8.6	2	0.6	<0.0001^†^	1.40	1.29–1.52
Skull/basilar skull	39	8.2	1	0.3	<0.0001^†^	1.42	1.32–1.53
Face	10	2.1	1	0.3	0.0293^†^	1.34	1.12–1.59
Spine^‡^	4	0.8	2	0.6	0.2971^†^	1.04	0.67–1.61
Traumatic brain injury	148	31.2	43	12.3	<0.0001	1.34	1.23–1.47
Severe	8	1.7	0	0.0	0.0239^†^		
Moderate	30	6.3	0	0.0	<0.0001^†^		
Mild	110	23.2	43	12.3	0.0002	1.23	1.12–1.36
Intracranial hemorrhage							
Subdural hemorrhage	22	4.6	0	0.0	<0.0001^†^		
Intraparenchymal hemorrhage	21	4.4	0	0.0	<0.0001^†^		
Subarachnoid hemorrhage	16	3.4	0	0.0	0.0001^†^		
Epidural hemorrhage	6	1.3	0	0.0	0.0415^†^		
ICU length of stay (LOS)							
≥1 days	26	5.4	0	0.0	<0.0001^†^		
≥2 days	21	4.3	0	0.0	<0.0001^†^		
Hospital LOS							
≥1 days	59	12.4	17	4.9	0.0002	1.22	1.09–1.36
≥2 days	49	10.3	9	2.6	<0.0001	1.27	1.14–1.41
Injury Severity Score							
≥15	13	2.7	0	0.0	< 0.0001^†^		
Disposition							
Home	460	97.0	350	100.0	0.0012	0.68	0.64–0.71
Rehabilitation	8	1.7	0	0.0			
Long term acute care	1	0.2	0	0.0			
Outcome							
Alive	471	99.4	350	100.0	0.1362		
Dead	3	0.6	0	0			

*Risk of the injury for longboarder compared with skateboarder patients, adjusted for age and sex.

^†^Based on Fisher's Exact Test.

^‡^Cervical spine (1), thoracic spine (3), and sacrum/coccyx (2).

**Table 4:** Types of patient treatment.

Intervention	Longboard		Skateboard		Chi-square
	(*n* = 474)		(*n* = 350)		
	Number	%	Number	%	
Conservative treatment (nonoperative)	427	90.1	330	94.3	0.0293
Orthopedic surgical	36	7.6	19	5.4	0.2184
Neurosurgical	10	2.1	0	0.0	0.0065

**Table 5:** Frequency of fractures and traumatic brain injury according to age for longboarders and also skateboarders.

Specific injuries	Longboard			Skateboard		Chi-Square
Age group	(*n* = 474)			(*n* = 350)		*P* value
	Number	%		Number	%	
Extremity fractures						
<15	10	47.6	0.7304	60	42.2	0.1055
15–24	74	39.0		37	31.9	
25+	107	40.7		28	30.4	
Upper extremity						
<15	7	33.3	0.4469	57	40.1	0.0004
15–24	41	21.6		25	21.6	
25+	64	24.3		18	19.6	
Radius/ulna/radial head						
<15	6	28.6	0.0128	41	28.9	0.0003
15–24	15	7.9		12	10.3	
25+	32	12.2		13	14.1	
Lower						
<15	2	9.5	0.7759	2	1.4	0.0080
15–24	16	8.4		11	9.5	
25+	18	6.8		9	9.8	
Tibia/fibula/ankle						
<15	1	4.8	0.9812	2	1.4	0.0470
15–24	8	4.2		9	7.8	
25+	12	4.6		5	5.4	
Traumatic brain injury (severe)						
<15	0	0.0	0.1819	0	0.0	
15–24	1	0.5		0	0.0	
25+	7	2.7		0	0.0	
Traumatic brain injury (moderate)						
<15	1	4.8	0.9057	0	0.0	
15–24	13	6.8		0	0.0	
25+	16	6.1		0	0.0	
Traumatic brain injury (mild)						
<15	3	14.3	0.0263	19	13.4	0.8640
15–24	56	29.5		13	11.2	
25+	51	19.4		11	12.0	

Note: percents were conditioned on age; that is, for each age, what percent experienced the specific injury.
